# Healthcare utilisation prior to the diagnosis of inflammatory bowel diseases and the influence of livestock exposure: A longitudinal case-control study

**DOI:** 10.1371/journal.pone.0195305

**Published:** 2018-04-09

**Authors:** Baukje J. van der Star, Christel E. van Dijk, Jan-Paul Zock, Lidwien A. M. Smit, Christos Baliatsas, Dick J. J. Heederik, C. Joris Yzermans

**Affiliations:** 1 Netherlands Institute for Health Services Research, NIVEL, Utrecht, The Netherlands; 2 Institute for Risk Assessment Sciences, IRAS, Utrecht University, Utrecht, The Netherlands; Universitatsklinikum Aachen, GERMANY

## Abstract

An increased prevalence of the inflammatory bowel diseases, ulcerative colitis and Crohn’s disease, was found amongst residents in a livestock dense area. We hypothesised that exposure to livestock farms might be a substantial environmental factor that contributes to the development of these diseases and that in the lead up to inflammatory bowel diseases potential risk factors can be identified. This study aimed to investigate the contribution of livestock exposure to the development of these diseases and the clinical events prior to the diagnosis. Electronic health records from 2006–2013 of general practices were used. The study population consisted of patients with a new diagnosis of inflammatory bowel diseases resident in areas with a high (n = 141) or lower (n = 109) livestock density. Patients with low back pain (n = 10,469) were used as controls. For those in a livestock dense area, distance to livestock farms was determined. Associations between morbidities and drug prescriptions in the reporting year and three years previous to the diagnosis, and the residential proximity to livestock exposure were investigated with multivariable logistic regression analyses. Acute and chronic morbidity of the gastrointestinal tract and associated drug prescriptions were predictive for the development of inflammatory bowel diseases. In addition, a positive association was found between infections and living within 500 meter of poultry farms and the development of inflammatory bowel diseases [OR: 3.3 (1.1–9.9)]. Nonetheless, overall livestock exposure contributed little to the development of these diseases. These results suggest that exposure to livestock farms on its own contributes minimal to the development of inflammatory bowel diseases. Nonetheless, having infections appeared to be a risk factor for neighbouring residents of poultry farms. More research is warranted to explain the increased prevalence of inflammatory bowel diseases amongst residents in areas with a high density of livestock.

## Introduction

Ulcerative colitis and Crohn’s disease are chronic inflammatory diseases of the gastrointestinal tract (GI-tract) and are known as inflammatory bowel diseases (IBD). Ulcerative colitis is characterised by inflammation of the mucosal layer of the colon and rectum. In contrast, Crohn’s disease is characterised by transmural inflammation causing strictures and fistulas, and may affect the entire GI-tract. Clinically, ulcerative colitis often manifests with abdominal pain and diarrhoea associated with blood, while Crohn’s disease often manifests with abdominal pain and constipation. However, in practice the distinction between these two diseases can be a challenge, resulting in diagnostic delays for months in adults up to several years in children [1,2]. Although ulcerative colitis and Crohn’s disease share similar features, such as a relapsing-remitting course, and although both affect young people, these diseases are considered to have different aetiological mechanisms [3]. Unfortunately, these mechanisms are still poorly understood. Nevertheless, it is presumed that the cause of IBD is multifactorial, including genetic mutations as well as lifestyle-related and environmental factors such as smoking habits and infections, respectively [4,5]. Interestingly, Heederik and Yzermans (2011) showed a higher prevalence rate of IBD in neighbouring residents of livestock farms in a rural area with a high density of these farms, as compared with those in areas with a much lower density of those farms [6]. This difference was confirmed in ongoing analyses investigating a longer period of time. This suggests that exposure to large-scale livestock farming, as an environmental factor, may contribute to the development of IBD, possibly by repeatedly stimulating mucosal tissue triggering an immune response [7]. On the contrary, others have shown that growing up on a farm is inversely associated with the development of juvenile IBD or IBD later in life [8–10]. Whether this also applies for neighbouring residents remains to be investigated.

As the diagnostic process of IBD can be challenging, longitudinal studies investigating the clinical events preceding this diagnosis are warranted. In one such study, Pimentel and colleagues showed that the clinical threshold of IBD-related symptoms were present on average seven years before diagnosis [11]. Whether the nature of symptoms preceding the diagnosis of IBD differs in light of exposure to livestock farms has not yet been investigated. We hypothesise that people exposed to a high density of livestock experience more often gastro-intestinal symptoms and appeal more often to the general practitioner. As every citizen in the Netherlands is obligatorily enrolled in the list of just one general practice, electronic health records of those practices can be used to investigate the medical history preceding the diagnosis of IBD. By combining this information with exposure estimates, we performed an observational study with a high quality of longitudinal registration of health records, including morbidity and drug prescriptions.

This study was designed to investigate whether livestock exposure contributes to the development of IBD and whether potential risk factors can be identified. To this end, we investigated clinical events preceding the diagnosis of IBD using health records in the reporting year and three previous years to the diagnosis of these diseases. These records were gathered for residents in a rural area with a high density of livestock farms and residents in rural areas with a much lower density of those farms, and findings compared to those gathered in a control group.

## Materials and methods

### Research design

A case-control study was performed using electronic health records (EHRs) of general practitioners (GPs) in the Netherlands. These records include morbidity and drug prescriptions at the patient level. Clinical events in the reporting year and three previous years to the diagnosis were investigated for patients with IBD, the cases, in comparison with patients with low back pain with or without radiating pain, the controls. Patients with low back pain were included as controls since these patients constitute typical general practice patients who also contact and visit the GP regularly, but their symptom patterns and associated healthcare utilisation are theoretically unrelated to IBD, as well as livestock exposure [12]. In addition, an area with a high density of livestock farms, so called exposed area, was compared with areas with a lower density of livestock farms, altogether referred to as the reference area. The exact specification of the exposed and reference area has been previously published [13,14]. Briefly, the exposed area included the Eastern part of the province Noord-Brabant and North-Western part of the province Limburg and is an area with the highest density of livestock farms in the Netherlands. The reference area included rural areas with mainly crop farming in the provinces of Noord-Holland, Zuid-Holland, Gelderland, Groningen, Overijssel and Utrecht. In both areas, cities with more than 30.000 residents were excluded to reduce the influence of urban air pollution.

### Ethical aspects

Research was performed in accordance with the Dutch Medical Treatment Act and Personal Data Protection Act. The protocol of the VGO study was approved by the Medical Ethical Committee of the University Medical Centre Utrecht. Privacy was ensured by using a Trusted Third Party (IVZ, Houten, The Netherlands), keeping medical information and address records separated at all times.

### Data availability statement

In consultation with the Medical Ethical Committee that approved the study protocol, data from the VGO study are not publicly available due to privacy protection of participants. The study's privacy regulations stated that only co-authors and researchers from NIVEL, IRAS, and RIVM (consortium partners) have access to the study database and researchers who are not part of the consortium partners will not have access to the study database since sharing an anonymized and de-identified dataset would still contain Electronic Health Records and the personal data of participants, which could potentially lead to the identification of subjects. Researchers may reach a data transfer and privacy agreement to access the data, which will include agreement on co-authorship with the consortium members who were responsible for designing the study and collecting the data. Researchers may contact the data access committee through Remco Coppen, PhD, LLM (r.coppen@nivel.nl) or non-personal departmental email (NIVEL: zorgregistraties@nivel.nl; IRAS secretary: Seeoh903@uu.nl), or contact Christos Baliatsas, PhD (c.baliatsas@nivel.nl), Lidwien Smit, PhD (l.a.smit@uu.nl) or Joris Yzermans, PhD (j.ijzermans@nivel.nl).

### General practices

Health records from GPs participating in the Netherlands Institute for Health Services Research Primary Care Database (NIVEL PCD) between 1 January 2006 and 31 December 2013 were used. The practices are representative of the Dutch GP population with respect to age, gender, region and urbanisation [15]. Furthermore, the NIVEL PCD has only small changes in the composition of practices between years. More information about the NIVEL Primary Care Database can be found at https://www.nivel.nl/en/node/4814. Following previous approaches [13], a number of a-priori inclusion criteria was used in terms of data completeness. More specifically, data on morbidity should be available for at least 65% of the contacts and for 46 or more weeks per year. Regarding the prescription data, drug prescriptions should have been registered in more than 85% of prescription records and for 46 or more weeks per year. As a result, 16 practices in the exposed area and 15 practices in the reference area were included from a total of 32 and 24, respectively.

### Patient selection

Following the selection of practices, incident patients with IBD and low back pain were identified according to their first registration of IBD or low back pain in their EHR. GPs registered the reason of contact, including diseases and presented symptoms, according to the International Classification of Primary Care (ICPC) [16]. In this classification, symptoms and diseases are given an unique code according to organ or system, as well as general/unspecified, psychological problems or social problems. The code consists of one letter followed by two numbers. The letter refers to the organ or system, the numbers 1–29 to symptoms and the numbers 70–99 to diseases. Incident IBD patients were identified by the ICPC-code D94, whereas incident patients with low back pain were identified by the ICPC-codes L03 and L86, without and with radiating pain, respectively. Patients with IBD and low back pain were excluded if EHRs were unavailable in the three years prior to their diagnosis. In addition, patients with low back pain with a registration of IBD were also excluded. Finally, when investigating potential associations between the development of IBD and variables of livestock exposure in the exposed area, presumed farmers were excluded as we were only interested in neighbouring residents. These were identified as patients living within 50 meters of livestock farm stables as shown by others [13,17].

### Morbidity and drug prescriptions

Health records in the reporting year and three previous years to the diagnosis were investigated for all patients using the ICPC-classification for morbidity and the Anatomical Therapeutical Chemical (ATC)-classification for drug prescriptions. In the ATC-classification, drug prescriptions are classified “according to the organ or system on which they act, and their therapeutic, pharmacological and chemical properties” [18]. In order to utilise a step-wise approach, both classifications were separately used to group morbidity or drug prescriptions in the reporting year and three previous years to the diagnosis into clusters and categories. Whereas the clustering of relevant ICPC-codes was adapted from Van Gils-Van Rooij et al. [19], clustering of the drug prescriptions was performed according to the guidelines for ATC classification and Defined Daily Dose assignment [18]. Morbidity included amongst others symptoms of the gastro-intestinal tract including diarrhoea and chronic diseases concerning the GI-tract but also autoimmune diseases and infections. Drug prescriptions were categorised for example in antidiarrheal and intestinal anti-inflammatory/anti-infective agents, corticosteroids and laxatives. Details of these clusters and categories, including the ICPC- and ATC-codes, are provided in S1 Appendix and S2 Appendix.

### Exposure to livestock farms

Detailed information on livestock farms was used to investigate the influence of livestock exposure to the development of IBD. The provinces of Noord-Brabant and Limburg provide mandatory licenses and therefore retain information among others on the number of livestock farms and type of animals, including pigs, poultry and cattle. As previously described, the distance between the patients’ residences in the exposed area and livestock farms was calculated by a geographic information system using the residential addresses and area codes (ArcGis 10.1; Esri, Redlands, CA, USA) [13,17,20]. Contrary to patients in the exposed area, computing individual exposure estimates for those in the reference area was not possible as we did not have information on their residential addresses. Subsequently, the following variables of exposure were determined in the exposed area: distance to nearest livestock farm, less than 500m versus more than 500m, the number of livestock farms within 500m and the presence of farm animals within 500m, all used as binary variables. When investigating the presence of farm animals (e.g. pigs) within 500m, the reference group comprised patients with no pigs or any other farm animals within 500m. Information on livestock exposure was used from 2012 [20].

### Data analysis

Differences in total healthcare utilisation for cases and controls between the exposed and reference area, including mean number of presented morbidity and drug prescriptions from all years together, was tested using the Mann-Whitney U test. Differences in gender and age at diagnosis between those areas as well as between cases and controls were tested using the Student’s *t* test.

In order to investigate potential associations between incidence of IBD and morbidity and drug prescriptions in the reporting year and three previous years to the diagnosis and to investigate whether the area of residence modifies these associations, logistic regression analyses were performed. Inflammatory bowel disease—‘1’ was IBD, ‘0’ was low back pain—was used as the dependent variable and the presence of the morbidity and drug prescription clusters were used as independent variables. Separate analyses were performed for each independent variable, and results were corrected for gender and age at diagnosis. Results were stratified for the area, and p-values for the multiplicative interactions (area × morbidity, area × drug prescriptions and area × exposure variable) are shown.

To investigate the influence of livestock exposure as an environmental factor to the development of IBD, logistic regression analysis was performed. Development of IBD was the dependent variable and livestock exposure the independent variable. Again, correction was performed for the potential confounders gender and age at diagnosis. Expanding on this analysis, the influence of livestock exposure variables, such as type of livestock, as effect modifiers was investigated in the exposed area. Results were stratified for the exposure variable, and p-values for multiplicative interactions (reference no animals) were determined from combined models.

Finally, given the hierarchical structure of the data, analyses were repeated on the basis of multilevel logistic regression to verify consistency of the results. Multilevel analyses showed similar results and are therefore not presented. Stata (version 13.1, StataCorp LP) was used to perform all analyses.

## Results

### Characteristics of the study population

**Fig 1:**
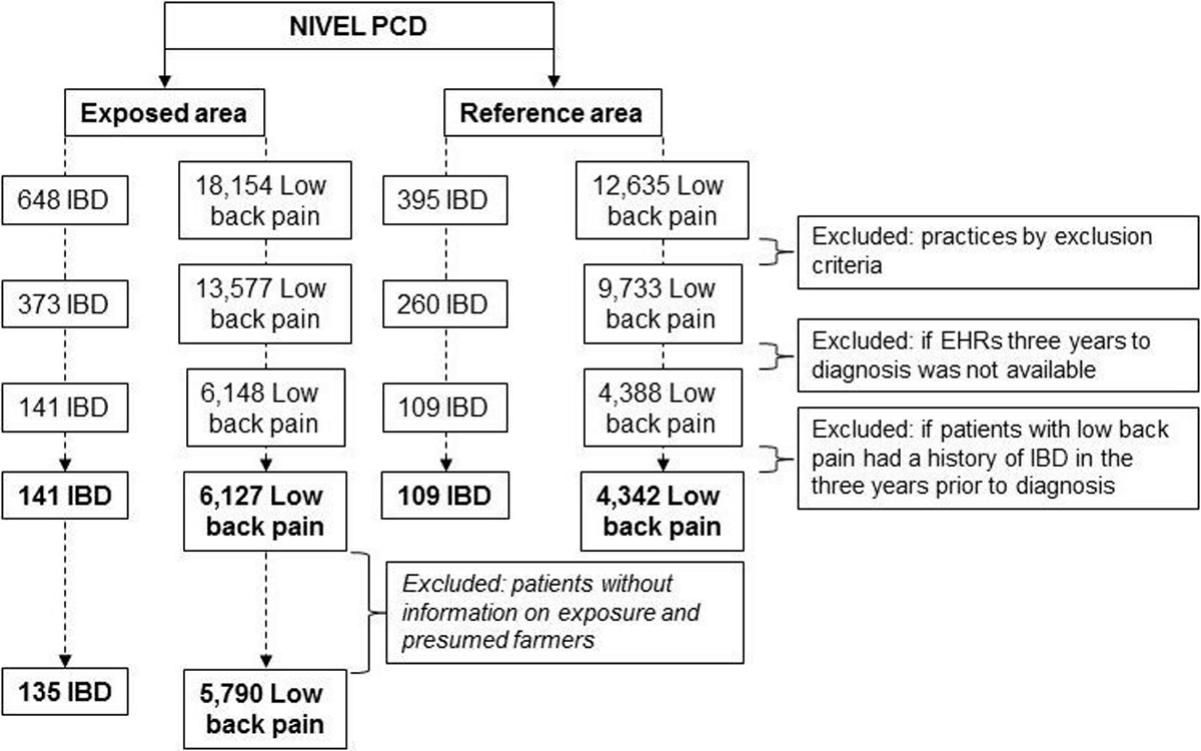
Selection of study population. Patients without information on estimated exposure and presumed farmers were excluded only for logistic regression analyses with livestock exposure variables. EHRs, Electronic health records; IBD, Inflammatory bowel disease; NIVEL PCD, Netherlands Institute for Health Services Research Primary Care Database.

Details of the study population are shown in Fig 1 and Table 1. In total, 250 patients with IBD and 10,469 patients with low back pain were selected (Fig 1). Of the 250 cases, 141 (56%) lived in the exposed area. Of the controls, 6,127 (59%) lived in the exposed area. Details with respect to estimated exposure to livestock farms were available for 135 cases and 5,790 controls. Controls did not differ significantly in age and gender from cases. Overall distribution of gender and age for cases was not statistically different between both areas. In contrast, controls in the exposed area were on average older than those in the reference area (p = 0.001, Table 1). Concerning the total healthcare utilisation in the reporting year and three previous years to the diagnosis, controls in the exposed area presented on average less often with morbidity and had less drug prescriptions than those in the reference area (p = 0.001 and p < 0.01, respectively). For cases, no significant differences in total healthcare utilisation between the areas were observed. Furthermore, 47% of the cases and 55% of the controls lived at a distance of 500m or less from the nearest livestock farm. Most of these patients lived within 500m of cattle (32% of cases and 43% of controls). Finally, almost 2% of cases and 4% of controls did not receive any drug prescriptions during the three-year period prior to diagnosis (data not shown).

**Table 1:** Characteristics of the study population.

Characteristic	Exposed area		Reference area	
	Inflammatory bowel disease	Low back pain	Inflammatory bowel disease	Low back pain
Patients, n	141	6,127	109	4,342
Gender (female)	54%	51%	48%	52%
Age, in years, mean (±SD)	50 (±17)	51 (±18)***	49 (±20)	50 (±19)
3–24, n (%)	11 (8%)	651 (11%)	20 (18%)	494 (11%)
25–44, n (%)	41 (29%)	1,406 (23%)	19 (17%)	1,126 (26%)
45–64, n (%)	58 (41%)	2,522 (41%)	41 (38%)	1,720 (40%)
≥65–104, n (%)	31 (22%)	1,548 (25%)***	29 (27%)	1,002 (23%)
**Healthcare utilisation**				
Total number of presented morbidity with GP in the reporting year and three previous years to the diagnosis, mean (±SD)	31 (±32)	29 (±31)***	38 (±42)	34 (±39)
Total number of drug prescriptions in the reporting year and three previous years to the diagnosis, mean (±SD)	43 (±65)	32 (±57)**	46 (±78)	34 (±55)
**Exposure to livestock**				
Patients, n	135	5,790	N/A	N/A
Distance to nearest livestock farm, n (%)			N/A	N/A
≤500m	64 (47%)	3,165 (55%)		
>500m	71 (53%)	2,625 (45%)		
Number of livestock farms ≤500m, n (%)				
0	72 (53%)	2,663 (46%)		
1–2	32 (24%)	1,810 (31%)		
3 > 13	30 (22%)	1,314 (23%)		
Presence of farm animals ≤500m (yes or no), n (%)				
Pigs	33 (24%)	1,508 (26%)		
Poultry	22 (16%)	642 (11%)		
Cattle	43 (32%)	2,465 (43%)		
Others (goats, sheep and minks)	25 (19%)	657 (11%)		

** p-value < 0.01

*** p-value = 0.001, ref are patients in the reference area

### Association between morbidity in the reporting year and three previous years to the diagnosis and incident IBD

In general, morbidity in the reporting year and three previous years to the diagnosis was positively associated with the development of IBD (Table 2). Irrespective of the area, experiencing acute somatic symptoms of the GI-tract was positively associated with the development of IBD [OR: 3.0 (95%CI: 1.6–5.7) in the exposed area and OR: 4.7 (95%CI: 2.1–10) in the reference area]. This was likewise for patients with chronic/long-term diseases of the GI-tract [OR: 1.9 (95%CI: 1.1–3.2) and OR: 3.4 (95%CI: 2.0–5.8), respectively, Table 2]. A history of autoimmune diseases in the reporting year and three previous years to the diagnosis was positively associated with the development of IBD, but only in the exposed area [OR: 3.1 (95%CI: 1.7–5.7)]. Nonetheless, this association was not statistically different between the areas (*p* for interaction = 0.25). In contrast, residents with infections of the GI-tract in the reference area were more at risk for development of IBD than those in the exposed area (*p* for interaction = 0.01, Table 2).

**Table 2:** Association between incidence of IBD and morbidity (diseases and presented symptoms) in the reporting year and three previous years to the diagnosis.

Morbidity (yes or no)	Exposed area		Reference area		P-value for interaction^c^
	n (%)^a^	OR (95% CI)^b^	n (%)^a^	OR (95% CI)^b^	
*Total acute somatic symptoms/MUPS*	130 (92%)	1.4 (0.8–2.7)	102 (94%)	2.1 (0.95–4.5)	0.53
Acute somatic symptoms: symptoms of the GI-tract	85 (60%)	3.0 (1.6–5.7)**	67 (61%)	4.7 (2.1–10)**	0.58
*Total infections*	88 (62%)	1.1 (0.8–1.6)	80 (73%)	1.7 (1.1–2.6)*	0.16
Infections of the eye	10 (7%)	1.3 (0.6–2.5)	11 (10%)	2.1 (1.0–4.2)*	0.35
Infections of the skin	39 (28%)	1.3 (0.8–1.9)	30 (28%)	1.6 (0.97–2.7)	0.51
Infections of the airways	48 (34%)	1.2 (0.8–1.8)	43 (39%)	1.7 (1.1–2.8)*	0.26
Infections of the GI-tract	8 (6%)	1.6 (0.7–3.3)	17 (16%)	6.3 (3.3–12)**	**0.01**
Infections of the urinary tract	19 (13%)	1.1 (0.6–2.0)	15 (14%)	1.4 (0.7–2.8)	0.82
*Total chronic/long-term diseases*	96 (68%)	1.3 (0.9–1.9)	74 (68%)	1.3 (0.9–2.1)	0.99
Chronic/long-term diseases of the GI-tract	29 (21%)	1.9 (1.1–3.2)*	33 (30%)	3.4 (2.0–5.8)***	0.23
Chronic/long-term diseases of the cardiovascular system	21 (15%)	1.3 (0.7–2.7)	25 (23%)	1.0 (0.5–2.0)	0.61
Chronic/long-term diseases of the airways	26 (18%)	1.5 (0.9–2.5)	19 (17%)	1.2 (0.7–2.3)	0.80
Chronic/long-term diseases of the skin	26 (18%)	1.2 (0.7–1.9)	22 (20%)	1.2 (0.7–2.2)	0.61
Chronic/long-term diseases: autoimmune diseases	24 (17%)	3.1 (1.7–5.7)***	11 (10%)	0.9 (0.4–2.2)	0.25
*Total neoplasms*	22 (16%)	0.8 (0.5–1.3)	20 (18%)	1.2 (0.7–1.9)	0.39
Neoplasms: cancer	7 (5%)	0.9 (0.4–1.9)	7 (6%)	1.3 (0.6–2.8)	0.50
*Acute psychological and social disorders*	36 (26%)	1.0 (0.7–1.4)	31 (28%)	1.0 (0.7–1.5)	1.00
Acute psychological and social problems	23 (16%)	1.0 (0.7–1.6)	18 (17%)	1.0 (0.6–1.6)	0.76
Anxiety and depression	13 (9%)	1.6 (0.9–2.9)	11 (10%)	1.7 (0.9–3.3)	0.89
*Lifestyle*: *weight and diet*	7 (5%)	0.6 (0.3–1.4)	9 (8%)	0.8 (0.4–1.7)	0.62

GI-tract, Gastrointestinal tract; IBD, Inflammatory bowel disease; MUPS, Medically unexplained physical symptoms

* p-value < 0.5

** p-value < 0.01

*** p-value < 0.001

^a^ Number and percentage of patients with IBD with respective morbidity in the reporting year and three previous years to the diagnosis

^b^ Adjusted for age at diagnosis and gender, ref: neighbouring residents without respective morbidity

^c^ P-value for interaction: area × morbidity

### Association between drug prescriptions in the reporting year and three previous years to the diagnosis and incident IBD

In general, drug prescriptions were positively associated with the development of IBD (Table 3). In particular, one or more prescriptions for antidiarrheal and intestinal anti-inflammatory/anti-infective agents in the reporting year and three previous years to the diagnosis was positively associated with the development of IBD [OR: 33 (95%CI: 22–48) in the exposed area and OR: 40 (95%CI: 26–62) in the reference area]. Laxatives were also positively associated with the development of IBD in both areas (Table 3). Furthermore, the positive association between drugs for functional GI-disorders and nasal preparations and the development of IBD for residents in the reference area were the only two associations that were significantly different compared with residents in the exposed area (*p* < 0.05 for interaction).

**Table 3:** Association between incidence of IBD and drug prescriptions in the reporting year and three previous years to the diagnosis^a^.

Drug prescriptions (yes or no)	Exposed area		Reference area		P-value for interactiond^d^
	n (%)^b^	OR (95% CI)^c^	n (%)^b^	OR (95% CI)^c^	
Drugs for functional GI-disorders	10 (7%)	1.2 (0.6–2.3)	19 (17%)	2.9 (1.7–4.8)**	**0.046**
Laxatives	59 (42%)	4.6 (3.3–6.6)**	48 (44%)	5.4 (3.6–8.1)**	0.65
Antidiarrheal, intestinal anti-inflammatory/anti-infective agents	66 (47%)	33 (22–48)**	54 (50%)	40 (26–62)**	0.53
Symptoms and diseases of alimentary tract and metabolism	64 (45%)	1.4 (0.99–2.1)	44 (40%)	1.1 (0.7–1.7)	0.42
Symptoms and diseases of the blood and blood forming organs	23 (16%)	3.0 (1.9–4.8)**	11 (10%)	2.0 (1.1–3.8)*	0.29
Cardiovascular diseases	56 (40%)	1.2 (0.8–1.8)	48 (44%)	1.5 (0.9–2.3)	0.56
Antifungals for dermatological use	28 (20%)	1.4 (0.9–2.2)	15 (14%)	1.0 (0.6–1.7)	0.29
Symptoms and diseases of the skin	64 (45%)	1.4 (0.99–2.0)	39 (36%)	1.1 (0.8–1.7)	0.39
Symptoms and diseases of the genito-urinary tract and reproduction	47 (33%)	1.5 (1.0–2.2)*	27 (25%)	1.1 (0.7–1.8)	0.21
Symptoms and diseases of the endocrine glands	32 (23%)	1.8 (1.2–2.7)*	21 (19%)	1.3 (0.8–2.2)	0.37
Antibacterial drugs for systemic use	74 (52%)	1.3 (0.9–1.9)	59 (54%)	1.3 (0.9–1.9)	0.76
Antiviral medication	29 (21%)	1.3 (0.9–2.0)	37 (34%)	1.8 (1.2–2.8)*	0.38
Immunosuppressant drugs excluding corticosteroids	11 (8%)	8.4 (4.3–17)*	10 (9%)	16 (7.5–34)**	0.21
Anti-inflammatory and anti-rheumatic drugs excluding corticosteroids	57 (40%)	0.5 (0.4–0.8)*	47 (43%)	0.5 (0.4–0.8)**	0.85
Symptoms and diseases of the musculoskeletal system	15 (11%)	2.5 (1.4–4.5)*	11 (10%)	2.1 (1.1–4.2)*	0.73
Analgesic drugs	28 (20%)	1.0 (0.6–1.5)	17 (16%)	0.7 (0.4–1.1)	0.27
Antidepressants (in combination with psycholeptics)	15 (11%)	1.2 (0.7–2.1)	17 (16%)	1.6 (0.95–2.8)	0.55
Symptoms and diseases of the nervous system	30 (21%)	1.1 (0.7–1.6)	28 (26%)	1.3 (0.8–2.0)	0.63
Drugs for obstructive airway diseases, excluding corticosteroids	26 (18%)	1.2 (0.8–1.8)	18 (17%)	1.2 (0.7–2.0)	0.94
Cough and cold drugs	19 (13%)	1.0 (0.6–1.6)	17 (16%)	1.0 (0.6–1.7)	0.97
Nasal preparations and antihistamines for systemic use	35 (25%)	0.9 (0.6–1.3)	43 (39%)	1.7 (1.2–2.6)*	**0.03**
Symptoms and diseases of the sensory organs	43 (30%)	1.2 (0.8–1.7)	25 (23%)	0.9 (0.6–1.4)	0.30

GI-disorders, Gastrointestinal disorders; IBD, Inflammatory bowel disease

* p-value < 0.5

** p-value < 0.01

^a^ Results are shown if drugs were prescribed for at least 10 patients with IBD

^b^ Number and percentage of patients with IBD with respective drug prescription in the reporting year and three previous years to the diagnosis

^c^ Adjusted for age at diagnosis and gender, ref: neighbouring residents without respective drug prescription

^d^ P-value for interaction: area × drug prescription

### Influence of livestock exposure on the development of IBD

Within the exposed area, an indication was found for an inverse association between the presence of livestock farms within 500m of the patients’ residences and the development of IBD. However, it did not reach conventional levels of statistical significance [OR: 0.7 (95%CI: 0.5–1.05), not in table]. The association with the number of livestock farms within 500m was in the same direction, with an odds ratio of 0.98 (95%CI: 0.9–1.1) per farm compared with residents who did not live within 500m of livestock farms in this area.

### Influence of livestock exposure on the association between morbidity in the reporting year and three previous years to the diagnosis and incident IBD

Positive and statistically significant associations between infections in the reporting year and three previous years to the diagnosis and the development of IBD were only found among those living within 500m of poultry farms, in particular for infections of the airways (*p* = 0.006 for interaction, Table 4). Furthermore, no positive associations were found between morbidity and the development of IBD for neighbouring residents living within 500m of cattle (data not shown). Finally, the associations between drug prescriptions and the development of IBD as shown in Table 3 were not modified by the exposure variables and are therefore not presented.

**Table 4:** Association in incident IBD patients between morbidity in the reporting year and three previous years to the diagnosis and farm animals ≤500m.

Morbidity (yes or no)	Farm animals ≤500m (yes or no)										
	No animals		All animals			Pigs			Poultry		
	n (%)^a^	OR (95% CI)^b^	n (%)^a^	OR (95% CI)^b^	P-value for interaction^c^	n (%)^a^	OR (95% CI)^b^	P-value for interaction^c^	n (%)^a^	OR (95% CI)^b^	P-value for interaction^c^
*Total acute somatic symptoms/MUPS*	70 (52%)	3.6 (0.9–15)	55 (41%)	1.1 (0.5–2.4)	0.19	31 (23%)	2.3 (0.5–9.8)	0.55	21 (16%)	2.8 (0.4–21)	0.85
Acute somatic symptoms: symptoms of the GI-tract	44 (33%)	6.5 (1.6–27)*	25 (19%)	2.6 (1.1–6.1)*	0.34	19 (14%)	4.9 (1.1–22)*	0.67	17 (13%)	7.1 (0.9–56)	0.97
*Total infections*	39 (29%)	0.7 (0.5–1.2)	43 (32%)	1.6 (0.9–2.7)	0.06	22 (16%)	1.3 (0.6–2.6)	0.26	18 (13%)	3.3 (1.1–9.9)*	**0.02**
Infections of the eye	4 (3%)	0.7 (0.3–2.1)	6 (4%)	2.2 (0.8–5.7)	0.16	3 (2%)	1.7 (0.4–6.5)	0.36	3 (2%)	6.0 (1.2–29)*	**0.047**
Infections of the skin	16 (12%)	0.8 (0.4–1.5)	20 (15%)	1.9 (0.99–3.6)	0.07	9 (7%)	1.4 (0.6–3.3)	0.35	8 (6%)	4.2 (1.2–15)*	**0.02**
Infections of the airways	20 (15%)	0.7 (0.4–1.3)	23 (17%)	1.7 (0.9–3.1)	0.07	12 (9%)	1.4 (0.6–3.2)	0.24	12 (9%)	4.5 (1.4–15)*	**0.006**
Infections of the GI-tract	4 (3%)	1.1 (0.4–3.2)	4 (3%)	2.4 (0.8–7.3)	0.33	3 (2%)	2.7 (0.7–10)	0.33	1 (1%)	3.5 (0.4–34)	0.39
Infections of the urinary tract	13 (10%)	1.3 (0.6–2.6)	5 (4%)	1.0 (0.4–3.0)	0.56	3 (2%)	1.2 (0.3–5.0)	0.62	2 (1%)	3.1 (0.4–24)	0.63
*Total chronic/long-term diseases*	52 (39%)	1.2 (0.7–2.2)	39 (29%)	1.3 (0.8–2.3)	0.39	19 (14%)	1.2 (0.6–2.5)	0.24	14 (10%)	1.2 (0.5–3.1)	0.70
Chronic/long-term diseases of the GI-tract	14 (10%)	1.4 (0.7–3.1)	14 (10%)	2.5 (1.2–5.1)*	0.96	8 (6%)	**3.3** (**1.3–8.5**)*	0.99	4 (3%)	1.8 (0.5–6.5)	0.75
Chronic/long-term diseases of the cardiovascular system	14 (10%)	1.1 (0.4–2.7)	6 (4%)	1.4 (0.5–4.3)	0.19	5 (4%)	1.9 (0.5–6.9)	0.51	4 (3%)	1.7 (0.4–8.1)	0.89
Chronic/long-term diseases of the airways	12 (9%)	1.3 (0.6–2.9)	12 (9%)	1.5 (0.7–3.1)	0.97	4 (3%)	0.8 (0.3–2.6)	0.39	4 (3%)	1.1 (0.3–3.9)	0.93
Chronic/long-term diseases of the skin	16 (12%)	1.3 (0.6–2.6)	8 (6%)	0.9 (0.4–2.0)	0.22	3 (2%)	0.6 (0.2–2.3)	0.14	2 (1%)	0.5 (0.1–2.7)	0.28
Chronic/long-term diseases: autoimmune diseases	14 (10%)	3.2 (1.4–7.1)**	8 (6%)	2.6 (1.0–6.7)*	0.38	4 (3%)	2.8 (0.8–9.9)	0.37	2 (1%)	1.1 (0.2–6.7)	0.42
*Total neoplasms*	13 (10%)	0.99 (0.5–1.8)	9 (7%)	0.8 (0.4–1.6)	0.46	6 (4%)	1.1 (0.4–2.7)	0.94	2 (1%)	0.5 (0.1–2.0)	0.33
Neoplasms: cancer	3 (2%)	0.6 (0.2–2.0)	4 (3%)	1.5 (0.5–4.5)	0.49	3 (2%)	3.3 (0.9–12)	0.27	0 (0%)	-	-
*Acute psychological and social disorders*	24 (18%)	1.2 (0.7–1.9)	12 (9%)	0.8 (0.4–1.6)	0.29	5 (4%)	0.6 (0.2–1.7)	0.20	4 (3%)	0.8 (0.3–2.4)	0.45
Acute psychological and social problems	15 (11%)	1.2 (0.7–2.2)	8 (6%)	1.0 (0.5–2.1)	0.49	1 (1%)	0.2 (0.03–1.6)	0.09	3 (2%)	1.1 (0.3–3.8)	0.69
Anxiety and depression	10 (7%)	2.3 (1.1–4.6)*	3 (2%)	1.0 (0.3–3.3)	0.18	2 (1%)	1.2 (0.3–5.2)	0.37	1 (1%)	1.1 (0.1–8.3)	0.48
*Lifestyle*: *weight and diet*	5 (4%)	0.8 (0.3–1.9)	2 (1%)	0.5 (0.1–2.0)	0.40	1 (1%)	0.6 (0.1–4.9)	0.55	1 (1%)	0.6 (0.1–4.7)	0.75

GI-tract, Gastrointestinal tract; IBD, Inflammatory bowel disease; MUPS, Medically unexplained physical symptoms

* p-value < 0.5

** p-value < 0.01

^a^ Number and percentage of patients with IBD with respective morbidity in the reporting year and three previous years to the diagnosis

^b^ Adjusted for age at diagnosis and gender, ref: neighbouring residents without respective morbidity

^c^ P-value for interaction: exposure variable × morbidity

## Discussion

The present study was designed to investigate whether livestock exposure contributes to the development of IBD and whether potential risk factors can be identified. Therefore the clinical events over a three-year period prior to established IBD diagnosis were explored. Morbidity of the GI-tract was confirmed to be highly associated with the development of IBD. In addition, having infections appeared to be a risk factor, but only in neighbouring residents living in the vicinity of poultry farms. Nonetheless, livestock exposure, as an environmental factor, contributed little to the development of IBD. These results support the hypothesis that residents in livestock dense areas generally are protected for the development of inflammatory diseases.

We investigated 141 cases in a livestock dense area and 109 cases in a reference area with less livestock farms, selected from electronic health records, as registered by GPs, of 648 and 395 patients with IBD, respectively. We found a positive association between other autoimmune diseases and the development of IBD. This positive association was only significant in the exposed area, irrespective of the presence of livestock within 500m. We considered the following autoimmune diseases: pernicious anaemia, diabetes mellitus type 1 and 2, psoriasis, multiple sclerosis and rheumatoid arthritis. For all these diseases, an association with IBD has also been found by others [21–23], which supports the hypothesis that patients with autoimmune diseases are prone to develop other autoimmune diseases [24]. However, shared aetiologies, which lie at the heart of most of these relations, may not fully explain why we only found this positive association for those in a livestock dense area. Further research is required to investigate the contribution of other environmental factors, such as particulate matter and microbial agents in this area, in more detail.

Our finding that infections, including respiratory infections, in the reporting year and three previous years to the diagnosis were positively associated with the development of IBD is in line with the literature [25–27]. This association was influenced by exposure to poultry farms. Although there is a lack of studies investigating the role of exposure to poultry in the development of IBD, associations between residential proximity to poultry and stimulation of mucosal tissue have been shown. These studies mainly focus on the respiratory system, showing increased exacerbations in patients with chronic obstructive pulmonary disease [12] and incidence of pneumonia in residents near poultry farms [13], suggesting irritation of mucosal cells by poultry-related emission. Furthermore, there have been suggestions of a possible shared pathological mechanism between respiratory diseases, such as asthma, and IBD (reviewed by Adar et al. [7]). This mechanism involves the increased recruitment of eosinophils, a specific type of innate immune cells, to the intestinal mucosa by the chemokine eotaxin-1. In patients with IBD, increased levels of this cytokine have been found in serum [28] and intestinal mucosal tissues [29,30]. In these latter two studies by Banks et al. (2003) and Coburn et al. (2013), tissue levels of eotaxin-1 correlated with disease activity. These results are in accordance with another study that showed a positive association between the number of eosinophils in the intestines of patients with IBD and disease severity [31]. Currently, a phase 2 clinical trial is being conducted with an antibody to eotaxin-1, called Bertilimumab, in patients with ulcerative colitis (ClinicalTrial.gov, NCT01671956). Altogether, these studies support the hypothesis that, besides their role in respiratory diseases, eosinophils may also play a role in the pathology of IBD. Whether their role is influenced by livestock exposure, requires further research. And as we only focussed on a three-year period before diagnosis, additional longitudinal studies should be performed to investigate whether patients may have presented with infections prior to this period.

In the current study, we confirmed some of the most typical symptoms and related manifestations that precede the diagnosis of ulcerative colitis and Crohn’s disease. For instance, patients with acute somatic symptoms of the GI-tract mainly reported abdominal pain, rectal bleeding, constipation and diarrhoea, as well as anal fissures and diverticulosis for chronic/long-term diseases of the GI-tract. The association between gastrointestinal morbidity and the development of IBD was also supported by the investigation of the drug prescriptions in the reporting year and three previous years to the diagnosis. Classic prescriptions for the treatment of these typical symptoms, such as laxatives, antidiarrheal and immunosuppressant drugs, showed a positive association with the development of IBD. This was also the case for drug prescriptions for anaemia. Being aware of false-positives, GPs might use these findings to achieve early recognition of IBD when they are presented with patients with these type of symptoms and manifestations. It is important to keep diagnostic delays to a minimum, as longer delays correlate with more severe disease and complications thereof [32]. In addition, others have shown that patients with Crohn’s disease benefit from treatment early in the disease course [33]. Nonetheless, reducing diagnostic delays is also dependent on the patients’ decision to visit the GP [32]. Identification of biomarkers and other factors involved in the development of IBD will further contribute to early recognition and management of IBD.

Our study was the first to investigate clinical events prior to the diagnosis of IBD in neighbouring residents of livestock farms, using health records from GPs in combination with estimated livestock exposure at the level of the patients’ home. Nonetheless, one source of weakness in this study is the small sample size, which could have affected the outcomes. This was mainly due to the criteria used to study the clinical events in the three years prior to diagnosis. Although the prevalence of IBD increased over the years, it should be mentioned that IBD is low-prevalent in the general population [34]. In addition, by limiting the medical history of patients for this period, potential risk factors that developed years before were therefore excluded. However, as shown by Hutfless et al., onset of Crohn’s disease can follow GI-tract infections within one year [27]. Other limiting factors are the lack of information on risk factors including smoking habits and family history, and other confounders such as the patients’ residential history and the patients’ occupation, which might be related to farms [35]. These factors and a family history of farming in neighbouring residents of livestock farms might explain why we found a negative association between development of IBD and living in an area with a high density of livestock farms [8,9]. Further research including questionnaires would be useful to take these confounders into account. In addition, whereas we had insight of individual exposure to livestock for residents in the exposed area, no information on residential addresses was available for those in the reference area. Finally, using the ICPC-code D94 did not allow us to make a distinction between ulcerative colitis and Crohn’s disease. Although these diseases have common features, they also show distinct characteristics. Therefore, future studies should aim to investigate these diseases separately. In contrast to these limitations, one of the major strengths of our study design was the selection of practices. Since every citizen in the Netherlands is obligatorily enrolled in the list of just one general practice, we could use a known denominator of the population. Furthermore, we only selected practices with a reliable quality of registration and did not base our selection on the number of patients or the location of the practice. This allowed us to use reliable data without selection bias. Another strong aspect of our study was its case-control design, including patients with low back pain as controls who showed a similar distribution of age and gender compared with the cases. Additionally, low back pain has no known relation with IBD or livestock exposure, yielding a control group that also contacts and visits the GP. Finally, other major strengths of this study were the use of detailed livestock exposure, although taken into account only a single calendar year, and the longitudinal study design. The latter strength allowed us to investigate risk factors for the development of IBD on the patient level.

In conclusion, this is the first study to investigate the clinical events preceding the diagnosis of IBD in neighbouring residents of livestock farms. Although we were not able to confirm overall livestock exposure as an environmental risk factor for the development of IBD, our results suggest that residents with infections who live in the vicinity of poultry farms, have an increased risk of developing IBD. Finally, this study contributes to the body of knowledge concerning the first symptoms prior to the diagnosis of ulcerative colitis and Crohn’s disease.

## Supporting information

S1 Appendix Morbidity. Clusters (in bold) and categories (in italic) of morbidity according to the ICPC-classification of symptoms and diseases [16].
(DOCX)

S2 Appendix Drug prescriptions. Drug prescriptions and clusters of drug prescriptions according to the guidelines for ATC classification and Defined Daily Dose assignment 2015 [18].
(DOCX)
