# Supplementary material for: Healthcare utilisation prior to the diagnosis of inflammatory bowel diseases and the influence of livestock exposure: A longitudinal case-control study
Source: PLoS One. 2018 Apr 9;13(4):e0195305. doi: 10.1371/journal.pone.0195305 (PMC5890991; doi:10.1371/journal.pone.0195305)
Supplement: S1 Appendix — Clusters (in bold) and categories (in italic) of morbidity according to the ICPC-classification of symptoms and diseases [16]. (DOCX) [file pone.0195305.s001.docx]

**Supporting information**

**S1 Appendix. Morbidity.** Legend Appendix S1. Clusters (in bold) and categories (in italic) of morbidity according to the ICPC-classification of symptoms and diseases [16].

**Total acute somatic symptoms/Medically unexplained physical symptoms (MUPS)**

A01-10, 14, 17, 29 B02-04, 29 D01-06, 08-22, 24-25, 29

F01-05, 13-18, 29 H01-05, 13, 15, 29, 81 K01-07, 29 L01, 02, 04-20, 29

N01-07, 16-19, 29 R01-09, 21-25, 29 S01-08, 20-24, 29

T01-08, 10-11, 29 U01-02, 04-07, 13-14, 29 X01-21, 29

Y01-08, 16, 29

*Acute somatic symptoms/MUPS: Symptoms of the GI-tract*

D01-06, 08-22, 24, 25, 29

**Total infections**

A70-78 B70, 71, 90 D70-73, 92

F70, 72-73 H70-74 K70, 71 L70

N70-73 R70-78, 80-83, 90 S09-11, 70-76, 84-85, 90, 95

T70 U70-72, 88

*Infections of GI-tract*

D70, 73

*Infections of the eye*

F70, 71, 73, 85

*Infections of airways*

R72, 74-83, 90

*Infections of skin*

S09-11, 70-76, 84-85, 90, 95

*Infections of urinary tract*

U71

**Acute psychological and social disorders**

P01-13, 20-25, 29, 74-79, 99 Z01-16, 18-25

*Acute psychological and social problems*

P01-04,06,20,77 Z05,11,12,24

*Anxiety and depression*

P74, 76, 79

**Total chronic/long-term diseases**

A12 B80-82 D89-93, 95-98

F91-95 H82-86 K74-87, 89-94

L83-85, 88-91, 94-95, 97-98 N86-89 R91, 93-96

S86-88, 91, 97 T81, 85-88, 90, 93 U95

X87-88

*Chronic/long-term diseases of the GI-tract*

D82-87, 91-93, 95, 98

*Chronic/long-term diseases of the cardiovascular system*

K86

*Chronic/long-term diseases of the airways*

R91, 95, 96, 97

*Chronic/long-term diseases of the skin*

S86-88, 91, 97

*Chronic/long-term diseases: diabetes*

T90

*Chronic/long-term diseases: autoimmune diseases*

B81 L88 N86 S91 T90

**Total neoplasms**

A79 B72-74 D74-78 F74 H75 K72 L71

N74-76 R84-86 S77-82 T71-73 U75-79 W72-73

X75-81 Y76-79

*Neoplasms: cancer*

A79 B72-74 D74-77 L71 N74 R84-85 S77 T71 U75-77 W72 X75-77 Y77-78

**Lifestyle: weight and diet**

T07, 08, 11, 93
