# Supplementary material for: Healthcare utilisation prior to the diagnosis of inflammatory bowel diseases and the influence of livestock exposure: A longitudinal case-control study
Source: PLoS One. 2018 Apr 9;13(4):e0195305. doi: 10.1371/journal.pone.0195305 (PMC5890991; doi:10.1371/journal.pone.0195305)
Supplement: S2 Appendix — Drug prescriptions and clusters of drug prescriptions according to the guidelines for ATC classification and Defined Daily Dose assignment 2015 [18]. (DOCX) [file pone.0195305.s002.docx]

**Supporting information**

**S2 Appendix. Drug prescriptions.** Legend Appendix S2. Drug prescriptions and clusters of drug prescriptions according to the guidelines for ATC classification and Defined Daily Dose assignment 2015 [18].

**Drugs for functional gastrointestinal disorders**

A03 Drugs for functional gastrointestinal disorders

**Laxatives**

A06 Laxatives

**Antidiarrheal, intestinal anti-inflammatory/anti-infective agents**

A07 Antidiarrheal, intestinal anti-inflammatory/anti-infective agents

**Cluster Symptoms and diseases of alimentary tract and metabolism**

A02 Drugs for acid related disorders

A05 Bile and liver therapy

A08 Anti-obesity preparations, excluding diet products

A09 Digestives, including enzymes

A10 Drugs used in diabetes

A11 Vitamins

A12 Mineral supplements

A16 Other alimentary tract and metabolism products

**Cluster Symptoms and diseases of the blood and blood forming organs**

B02 Anti-haemorrhagic drugs

B03 Anti-anaemic drugs

B05 Blood substitutes and perfusion solutions

**Cluster Cardiovascular diseases**

B01 Antithrombotic agents

C01 Cardiac therapy

C02 Antihypertensive drugs

C03 Diuretic drugs

C04 Peripheral vasodilators

C05 Vasoprotective drugs

C07 Beta blocking agents

C08 Calcium channel blockers

C09 Agents acting on the renin-angiotensin system

C10 Lipid modifying agents

**Antifungals for dermatological use**

D01 Antifungals for dermatological use

**Cluster Antibiotics for dermatological use, antiseptics and disinfectants drugs**

D06A Antibiotics for dermatological use

D08 Antiseptics and disinfectants drugs

**Cluster Symptoms and diseases of the skin**

D02 Emollients and protectants

D03 Treatment of wounds and ulcers

D04 Antipyretics drugs

D06B, D06C Chemotherapeutics for topical use

D07 Topical dermatological corticosteroids

D09 Medicated dressings

D10 Acne drugs

D11 Other dermatological drugs

**Cluster Symptoms and diseases of the genital-urinary tract and reproduction**

G01 Gynaecological anti-infectives and antiseptics

G02 Other gynaecological drugs

G03 Reproductive hormones and modulators of the genital system

G04 Urological drugs

**Cluster Symptoms and diseases of the endocrine glands**

H01 Pituitary and hypothalamic hormones and analogues

H02 Corticosteroids systemic

H03 Thyroid therapy

H04 Pancreatic hormones

H05 Calcium homeostasis

L02 Endocrine therapy

**Antibacterial drugs for systemic use**

J01 Antibacterial drugs for systemic use

**Cluster Antiviral medication**

J05 Antiviral drugs

J06 Immune sera and immunoglobulins

J07 Vaccines

**Antineoplastic drugs**

L01 Antineoplastic drugs

**Immunosuppressant drugs excluding corticosteroids**

L04 Immunosuppressant drugs excluding corticosteroids (H01)

**Anti-inflammatory and anti-rheumatic drugs excluding corticosteroids**

M01 Anti-inflammatory and anti-rheumatic drugs excluding corticosteroids (H01)

**Cluster Symptoms and diseases of the musculoskeletal system**

M02 Topical products for joint and muscular pain

M03 Muscle relaxants

M04 Anti-gout preparations

M05 Drugs for treatment of bone diseases

M09 Other drugs for disorders of the musculoskeletal system

**Analgesic drugs**

N02 Analgesic drugs

**Hypnotics and sedatives**

N05C Hypnotics and sedatives

**Cluster Antidepressants (in combination with psycholeptics)**

N06A Antidepressants

N06CA Antidepressants in combination with psycholeptics

**Cluster Symptoms and diseases of the nervous system**

N01 Anaesthetic drugs

N03 Anti-epileptics

N05A Antipsychotics

N05B Anxiolytics

N04 Anti-parkinson drugs

N06B Psychostimulants, agents used for ADHA and nootropics

N06CB Psychostimulants in combination with psycholeptics

N06D Anti-dementia

N07A, N07B, N07X Other nervous system drugs, excl. anti-vertigo preparations (N07CA)

**Cluster Infections with protozoa, helminths and parasites**

P01 Antiprotozoal drugs

P02 Anthelmintic drugs

P03 Ectoparasiticides, including scabicides, insecticides and repellents

**Drugs for obstructive airway diseases, excl. corticosteroids**

R03 Drugs for obstructive airway diseases, excl. corticosteroids (H02)

**Cough and cold drugs**

R05 Cough and cold drugs

**Cluster Nasal preparations and antihistamines for systemic use**

R01 Nasal preparations

R06 Antihistamines for systemic use

**Cluster Symptoms and diseases of the sensory organs**

S01 Ophthalmological drugs

S02 Otologicals
